# Odontological Society of Pennsylvania

**Published:** 1893-01

**Authors:** 

**Affiliations:** Odontological Society of Pennsylvania


### ODONTOLOGICAL SOCIETY OF PENNSYLVANIA.

The regular meeting was held November 13, 1892, at the rooms of the Society, 1228 Walnut Street, Philadelphia, Dr. Louis Jack presiding.

After the transaction of the usual routine business, the discussion of the evening took place on the question, "Should Examining Boards have the Power to grant Certificates to Undergraduates?"

The subject was opened by a paper from Dr. Jack.

(For Dr. Jack's paper, see page 10.)

*Dr. Peirce.*—I rather hesitate to express my views on this subject, as it is a matter of some delicacy, inasmuch as I am connected with a school, and it may be somewhat warped with my associations. There is one thing we need to remember, and that is, not to confuse incompetent Examining Boards with competent ones.

My views are that competent Examining Boards should be thoroughly prepared to examine and say whether the applicant is qualified to practise dentistry. If we have these, they are for the purpose of judging of the qualification of applicants. They have no right, in my estimation, appointed as they are by the Legislature, to ask the applicant whether he has a diploma, or where he got his education. All they want to do is to ascertain if the applicant is qualified to practise dentistry, and it matters not where that qualification is obtained so long as he possesses it. While I have that feeling, still I concur in the sentiments of the paper as to disqualification of applicants who have come up before the board; yet the fact still remains that the law has no right to ask the question, where the information was gained. It is simply whether the information is possessed by the individual, and I would like our board so educated and so thoroughly appreciating their position that they should hold that ground, and not accept a diploma as a guarantee, and not accept anything but a thorough examination and satisfactory qualifications, and when we have that the public is protected, and we have an advance made in education.

I concur fully with the opinion expressed that applicants for examination are invariably disqualified. I have no hesitation in saying that, and that three years in college would not be too much instruction to require, but the fact still remains that the Legislature, in passing a law and the State in filling the Examining Boards should make those appointments with the view to the Examining Board being qualified, thoroughly qualified and conscientious, and if they were all like our president I should have no fear in the matter.

*Dr. Kirk.*—This is one of the subjects, I must confess, in which I have only a limited interest. This centres so much in other directions that I have not sufficient to go around.

If I understand correctly the statements made on this subject, that in the effort to obtain State legislation regulating the practice of dentistry, the committees have been met by the problem, the relation of State Examining Boards to college Faculties. The Constitution of the United States and the laws of the various States are repugnant to anything that looks like class legislation, and a law which would prohibit any man from practising dentistry, unless he was a regular graduate of a dental institution, would savor of that idea. Dr. Peirce has very clearly shown that when he states that such legislation would inhibit the class who wish to become self-educated. As a matter of principle, I don't see any objection to saying whether a man should not qualify himself to practise dentistry. The question is, Can he do it? It is the function of the State Examining Boards to answer that question.

I was on that board for two years, and had charge of the chemical and metallurgical examinations, and I know that the men who came up were absolutely unqualified in the most ordinary way, and certainly unfit to practise dentistry if they were to be gauged by that standard.

The State examination, if it is properly conducted, should be the barrier to the entrance of incompetent men into dentistry. Whether we should or should not examine men who have not passed a college examination, it seems to me hinges on that point of the law in respect to class legislation.

*Dr. Darby.*—It seems to me the injury is to the student himself. It is not unusual for a man to enter college, and, after he has received a smattering of dental education, he concludes to continue the work at home. He practically says, I can work with my preceptor, act as an assistant, can study a little, and, putting what I have learned in college with what I can learn at home, I can manage to pass this Examining Board. He perhaps succeeds in this, and enters into practice, but he has not that broad culture, that many-sidedness that he would have had if he had remained two years more at college, so that injury reflects upon himself, and he is the sufferer.

Now, that is practically the main question. I was eight or ten years with the Examining Board, and among the applicants were men who had attended one course of lectures. They were, perhaps, well up in some of the branches, were really quite well qualified in operative dentistry, so far as answering questions. It was usually upon that branch that I examined, but they were not, and never would be by home study, what they would have been if they had attended one or two years more at college. So I believe the State Board, or the certificates granted, are an injury to the men themselves, and if we were to prevent that manner of granting certificates by State Boards, numbers of men would be better off in the end. Those who want to study and enter the profession will always find a way, and while it may seem almost impossible to raise the money, it can always be done if they are in earnest. Therefore I am opposed to the State Board.

*Dr. Guilford.*—The remarks of Dr. Peirce pleased me very much, and he covered, I think, the ground very thoroughly. This is an important question. As the law now stands in various States,—our own among the number,—the State Board is required to examine any applicant, and if he be found qualified to practise, they must give him a certificate to that effect. The question is, whether this shall be continued or whether the law should be changed. Dr. Jack has given several reasons why the law should be changed, and there is something to be said on both sides. The student, at present being required to take a three years' course before graduation, finds at the end of the second year, perhaps, his funds are exhausted, and he is not able to continue through the next course. The question, then, is, Shall he have the privilege of practising a while in order to raise funds to complete his education? The only way of doing that is to appear before the board and pass the examination. If he is prepared to pass, if he correctly answers the required number of questions, I cannot see why he should not be allowed to practise. The board stands as a guard to the public against incompetency. If the man is not competent, it is the duty of the board to find that out and reject him; but if they cannot reject him, certainly, I think, they must give him the certificate. I fail to see any fault to be found with that.

Another question is, Is it not true, or probable, that a student, after having attended two full courses in a reputable dental college, is better qualified to practise than many men who have passed the board in the past? If a student of that kind is qualified, and has been in attendance at two full courses of lectures, and passes the examination before the board, I cannot see why he should not be admitted to practice. I am willing, so far as I am concerned, that the law shall be changed in that respect. I fail to see any real objection to it.

As to Examining Boards, we have them of all sorts. That is proved by the fact that before certain boards it is almost impossible for any man to pass. Some of them have rejected men who were considered by the colleges very well qualified. That has happened time and again. On the other hand, students who have only attended a single course in college have passed the boards in other States. Two in our own college, and one the year before, went home at the end of the first course, and, desiring to practise during the summer, went before the State Board and passed the examination successfully. Now, that shows, I think, that the men who examined them were incompetent to do so, and it shows us also that there is the greatest degree of difference between the State Boards.

Now, another point, and that I think was in the mind of the essayist to-night, and that is that perhaps a student who had attended one or two courses of lectures and had appeared before a State Examining Board, and had passed that Board, would not complete his education in college. There is a probability of that taking place, but I have never known it to occur. In the cases under my observation they have simply passed the board for the purpose of practising a certain time, and then continued their course; so it has not acted injuriously in that respect.

It is very well for us to have laws regulating the practice of dentistry, but it seems to me we ought to go back of that and have something to say in relation to the composition of the boards,—what sort of men shall be in them, and what their proficiency shall be for examination, what the requirements shall be and the way of examining, and that it should be somewhat uniform and a method of comparison between the boards. It seems to me that as it is now done it is very loose. The colleges make strict requirements, and more restrictions are being placed upon them each year, while with the Examining Boards there is no restriction.

There is another question, that of examining men who hold diplomas. That has hardly come up. The point I want to make is, in the first place, under the law as it now stands, different boards are required to examine any one who comes before them, and it seems to me it is not working any special harm. If a student is qualified to pass an examination, he will certainly inflict no injury upon the public.

*Dr. Truman.*—I find very little in this question. I have been astonished at the first and last speech. Here is a dean who is not satisfied with the teaching of his own school. He wants the diploma discredited by having the board re-examine the students. It is astonishing to me. I do not care to have any board examine a graduate of the University of Pennsylvania. I question their ability to do it. I am opposed to this granting of certificates, and I am surprised that my friend, Dr. Guilford, should defend a practice that will certainly end in discrediting dental education. It would be better to return to the old plan of every man studying in private and securing a certificate from his preceptor. The majority of boards may or may not be good.

This plan of granting certificates in some States has superseded the diploma. In a recent contest in New York State, in granting honorary degrees, it was asserted that a degree in New York State was superior to a diploma. If it has come to that, that a certificate issued by the State is superior to that granted by a college, we might with propriety give up all college instruction. A man can go to New York State and get his degree, and can then become a professor. And why not under the law? Is that an advance in education? I think not. I am opposed to the whole thing.

*Dr. Peirce*, in reply to Dr. Truman, said: The question under discussion is not whether incompetent boards should have the power to examine students. It is whether Examining Boards shall have power to grant certificates of qualification to undergraduates. We have to presume that the board has been legally appointed and has the qualification. We have to assume that in discussing the question. It is, simply, should Examining Boards have power to examine? If they have been appointed by the Legislature for the purpose of protecting the public, what else should they do? There is nothing else to say. They must have power to grant certificates, or else they should not be appointed. It is not a discussion of whether they are qualified. I should say, with Dr. Truman, there was not a fourth of them qualified to grant certificates, but if they are selected, it is their function, and, having been appointed, they have that power.

*Dr. Guilford.*—I am afraid I didn't make myself entirely clear. I judge so from what Dr. Truman has said. He seemed to catch the idea that I was in favor of the old method of examining a lot of men,—anybody at all,—and passing a good many of them and allowing them to practise without a regular dental education at the colleges. My idea is that it would work no harm as things are now constituted. The Examining Board of to-day in the different States is very different from ten years ago. They have learned a great deal, and their examinations are of a very different character in many of the States. Now, if a student or any one else comes before an Examining Board, it is their duty to find out if he is qualified, and if not, to reject him, and that is the end of it. If he is qualified, I don't see how he can be prevented from practising. I think it is the reverse of preventing men from going to college.

In the early days persons who wanted to practise would appear before the Examining Board, and if not qualified they would be rejected, and they knew they could not possibly practise in the State; the only thing to do was to go to college, and this they did. Dr. Peirce will remember that was the regular course men took. They found they could not pass the Examining Boards and they qualified themselves properly in colleges, and I think that is exactly the effect it would have to-day, and I am sure the examinations are more stringent than they were then. I would not be in favor of men entering the profession outside of the schools; in fact, I am in favor of a law requiring them to pass through a school and obtain a diploma, but at the same time I see no harm in allowing students to pass between the courses.

*Dr. Darby.*—I think what Dr. Guilford has said is partly true and partly wrong. He says that men who pass the examination before the board do not come back to college. That very result, in my opinion, injures the man. If he succeeds in passing the Examining Board he goes into practice without that culture he would get from a second or third course in college, and for that reason I am opposed to it.

*Dr. Jack.*—I might state a subject that will be of interest. In the earlier days we experienced as a result of our work very considerable complaint in reference to the number of men who received certificates of qualification from the board, and a resolution was passed at the State Association in which it was asked to appoint a committee for conference with the Board of Examiners to see if some means might not be devised by which a certificate of qualification would be made more difficult of attainment. After that two new members were put upon the board who were pledged to a strict examination, and when the board made up its organization the president announced that the incoming of the board meant a complete change in the policy of the board, and that certificates of qualification should not be granted unless they were most clearly deserved, and rules were adopted which made it very much more difficult to secure a certificate.[1]

While I am on the floor, I might state that a few words are necessary on my part to clear up one part of the argument. It is certainly true that in all these twenty-eight States the boards are obliged to examine all who come before them. The question brought before us here is, though, Should the boards have that power? Should the law give that power to the boards? would possibly have been a better expression to have given the question. Should the law confer upon Boards of Examiners power to grant certificates without college qualification?

J. Atkinson McKee, D.D.S.,
*Editor Odontological Society of Pennsylvania.*

[1] From 1876 to 1885, inclusive, twenty certificates were granted by the Pennsylvania Board. From 1886 to 1892 two were granted. This difference indicates the advanced ideas of the board.

